# Breast Ductal Carcinoma in Situ: Morphologic and Kinetic MRI Findings

**DOI:** 10.5812/iranjradiol.4876

**Published:** 2013-05-20

**Authors:** Mirjan M. Nadrljanski, Biljana B. Marković, Zorica Č. Milošević

**Affiliations:** 1 Department of Diagnostic Imaging, Institute of Oncology and Radiology of Serbia (IORS), Belgrade, Serbia; 2 Center for Radiology and Magnetic Resonance Imaging, Clinical Center of Serbia (KCS), Belgrade, Serbia; 3 Department of Radiology, Faculty of Medicine, University of Belgrade, Belgrade, Serbia

**Keywords:** Carcinoma, Intraductal, Non infiltrating, Magnetic Resonance Imaging, Breast Neoplasms, Image Enhancement, Gadolinium DTPA

## Abstract

**Background:**

Adequate diagnosis of ductal carcinoma in situ (DCIS) could lead to efficacious treatment. Due to the fact that DCIS lesions can progress to invasive carcinomas and that the sensitivity of the standard examination – mammography – is between 70 and 80%, use of a more sensitive diagnostic tool was needed. In detection of DCIS, contrast-enhanced magnetic resonance imaging (CE-MRI) has the sensitivity up to 96%.

**Objectives:**

Morphological features and kinetic parameters were evaluated to define the most regular morphological, kinetic and morpho-kinetic patterns on MRI assessment of breast ductal carcinoma in situ (DCIS).

**Patients and Methods:**

We retrospectively assessed eighteen patients with 23 histologically confirmed lesions (mean age, 52.4 ± 10.5 years). All patients were clinically and mammographically examined prior to MRI examination.

**Results:**

DCIS appeared most frequently as non-mass-like lesions (12 lesions, 52.17%). The differences in the frequency of lesion types were statistically significant (P<0.05). The following morphological patterns were detected: A: no specific morphologic features, B: linear/branching enhancement, C: focal mass-like enhancement, D: segmental enhancement, E: segmental enhancement in triangular shape, F: diffuse enhancement, G: regional heterogeneous enhancement in one quadrant not conforming to duct distribution and H: dotted or granular type of enhancement with patchy distribution. The difference in the frequency of the proposed patterns was statistically significant (P<0.05). There were eight lesions with mass enhancement, and six with segmental lesions: regional and triangular. There was no statistically significant difference in the frequency of enhancement curve types (P>0.05). There was no significant difference in the frequency of morpho-kinetic patterns.

**Conclusion:**

Non-mass-like lesions, lesions with focal or segmental distribution, with a “plateau” enhancement curve type were the most frequent findings of DCIS lesions on MRI.

## 1. Background

Ductal carcinoma in situ (DCIS) is the noninvasive form of breast cancer. It is, the clonal proliferation of malignant epithelial cells with no histological confirmation of basal membrane invasion (1). Typical manifestation includes breast calcifications mammographically apparent in up to 90%of the lesions (2). Ten to twenty percent of DCIS lesions appear as masses, architectural distortions or without calcifications (3) and 14-75% of DCIS lesions progress to invasive carcinoma. Adequate diagnosis provides the efficacious treatment of these lesions (4). Calcifications are not present in all DCIS lesions, lowering the sensitivity of mammography to 70-80% (5). Contrast-enhanced magnetic resonance imaging (CE-MRI) shows high sensitivity in DCIS detection (77-96%) (6-8). DCIS is considered as the direct precursor of invasive carcinoma usually in the same quadrant (9, 10). Classification of DCIS, which is based on morphologic, cytonuclear and architectural criteria, recognizes three categories of DCIS; poorly differentiated DCIS with the highest risk of stromal invasion; intermediately differentiated DCIS and well-differentiated DCIS with no evidence of necrosis (11). During tumor growth, the insufficient diffusion of oxygen and nutrients leads to hypoxic changes, necrosis and calcification (12). Enhancement of signal intensity (SI) is lower in DCIS compared to invasive carcinoma, explaining the low frequency of the “washout” curve type in DCIS.CE-MRI detects mammographically occult and multifocal lesions (13). On T2W sequence, DCIS is isointense or hypointense. SI enhancement frequently presents as the plateau curve (14). The intralesional contrast uptake is clumped with confluent areas, while the heterogeneous type appears less frequently (1). Calcifications are not identified by MRI exam, except the larger ones, perceived as the signal void (15). DCIS regularly appears as clumped enhancement of segmental or linear distribution (16). Esserman et al. analyzed 100 histologically confirmed DCIS lesions and defined the type of SI enhancement and distribution of lesions as focal non-mass-like; linear ductal; segmental, triangular enhancement (apex towards the nipple); regional single quadrant enhancement and multiregional enhancement (patchy or diffuse in two or more quadrants). According to a study conducted by Esserman et al. (17), the following patterns of internal contrast enhancement exist in DCIS: heterogeneous (non-uniform, between the regions of non-enhancement), clumped and homogeneous. Neubauer et al. proposed the classification of 5 MRI morphologic patterns (18). We proposed the following 8 morphologic patterns, based on enhancement and distribution, summarized in Figure 1 (17, 18).

**Figure:**
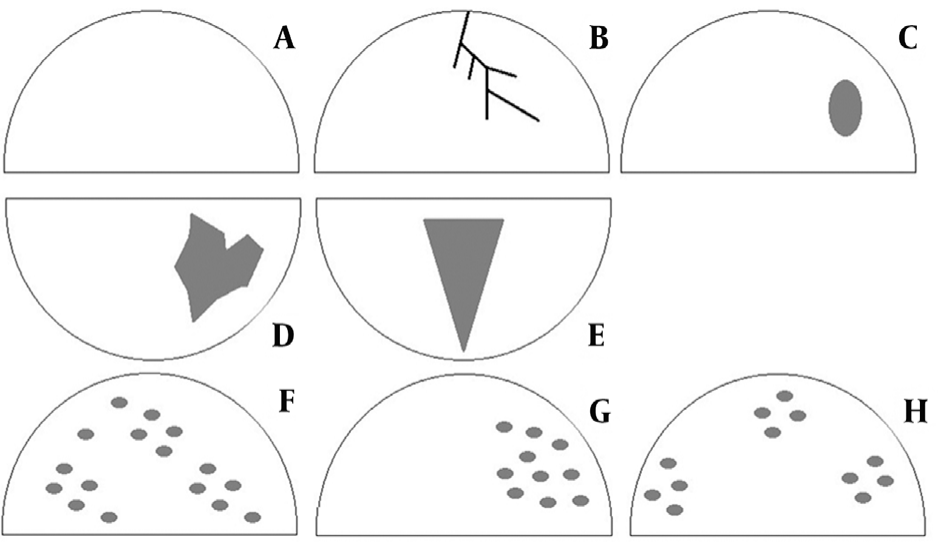
Figure 1. Categorization of morphologic types based on patterns of enhancement and distribution

Gadolinium (Gd-DTPA) is the extracellular contrast agent distributed in the vascular and then in the interstitial compartment (6). The contrast perfusion rate from blood vessels into the interstitial compartment increases with the grade of malignancy and the vascular density (1). The risk of malignancy depends on the kinetic pattern, ranging from 6% in type I and 64% in type II to 87% in type III (6, 7, 16-18). Rapid uptake is most often associated with DCIS in the initial phase, while all three types of curves are seen in the delayed phase, of which the plateau type is the most common (15, 16).

## 2. Objectives

The objective was to analyze the morphology and kinetic features of DCIS to define the most frequent morphologic and kinetic patterns.

## 3. Patients and Methods

The patients were examined in the Institute of Oncology and Radiology of Serbia, Belgrade from May 2009 to October 2011. We reviewed 832 MR-examination records of patients who were clinically examined, then evaluated by mammography and finally assessed by MRI. Twenty-three lesions were confirmed as DCIS. Taking into consideration the retrospective research with its limitations – the selection bias, we introduced the inclusion/exclusion criteria in order to narrow the group to patients with no genetic burden, no invasive breast carcinoma and all with histological confirmation – the surgical core biopsy being performed after breast MRI.All examinations were performed on a 1.5 T unit (Siemens Avanto®, Siemens Medical, Erlangen, Germany) with bilateral breast scans, including TSE and T2W sequences with and without fat signal suppression and 3D FLASH in the axial plane, before and after contrast injection (slice thickness, 2 mm; repetition time, 8.1 ms; echo-time, 4 ms;and flip-angle, 20 degrees). After the initial acquisition, the contrast agent Gd-DTPA (Magnevist®; Schering, Berlin, Germany) was administered intravenously, at a dose of 0.1 mM/kg body weight at a rate of 1mL/s, followed by the injection of a 10 mL saline flush.Statistical analysis was performed by chi-square and Fisher`s exact tests. A p value less than 0.05 was considered significant.

## 4. Results

In the series of 23 lesions, there were 10 mass lesions (43.5%), 12 non-mass-like lesions (52.2%) and one focus (4.3%) that was compatible with other studies mentioning non-mass-like lesions as the most frequent DCIS lesions (Table 1). The statistically significant difference was found in the frequency of the proposed patterns (P<0.05) and the C morphologic pattern was the most common.

**Table 1.** Distribution of Morphologic Patterns in the Lesions

Morphologic Pattern	Lesions, No.	Lesions, %
A – No Specific Morphologic Features	0	0
B – Linear/Branching Enhancement (Ductal Distribution)	4	17.4
C – Focal Mass-Like Enhancement	8	34.8
D – Segmental Homogeneous Enhancement	3	13
E – Segmental Enhancement in Triangular Shape (The Apex Pointed Towards the Nipple)	3	13
F – Diffuse Enhancement in at Least Two Quadrants	2	8.7
G – Regional Heterogeneous Enhancement in One Quadrant (Not Conforming to Duct Distribution)	3	13
H – Dotted/Granular Type with Patchy Distribution	0	0
Total	23	100

The plateau curve type was seen in 13 lesions (56.5%), followed by the continuous type that was detected in six lesions (26%) and the washout type observed in four lesions (17.4%). The difference between the frequency of curve types was not significant statistically (P>0.05). Curve types suspicious of malignancy; namely, plateau and washout were found in 17 of the lesions (approximately 74%) (Figures 2 and 3).

**Figure:**
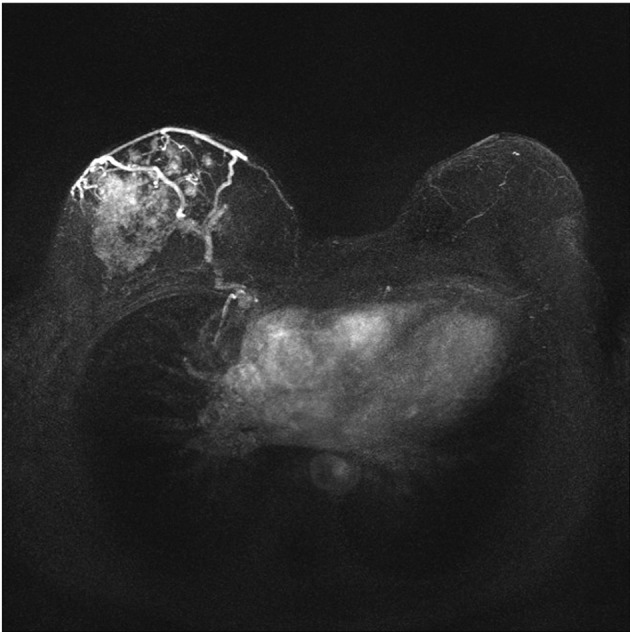
Figure 2. MRI in DCIS of the right breast demonstrating a non-mass-like enhancement using MIP (Maximum intensity projection) in post-processing

**Figure:**
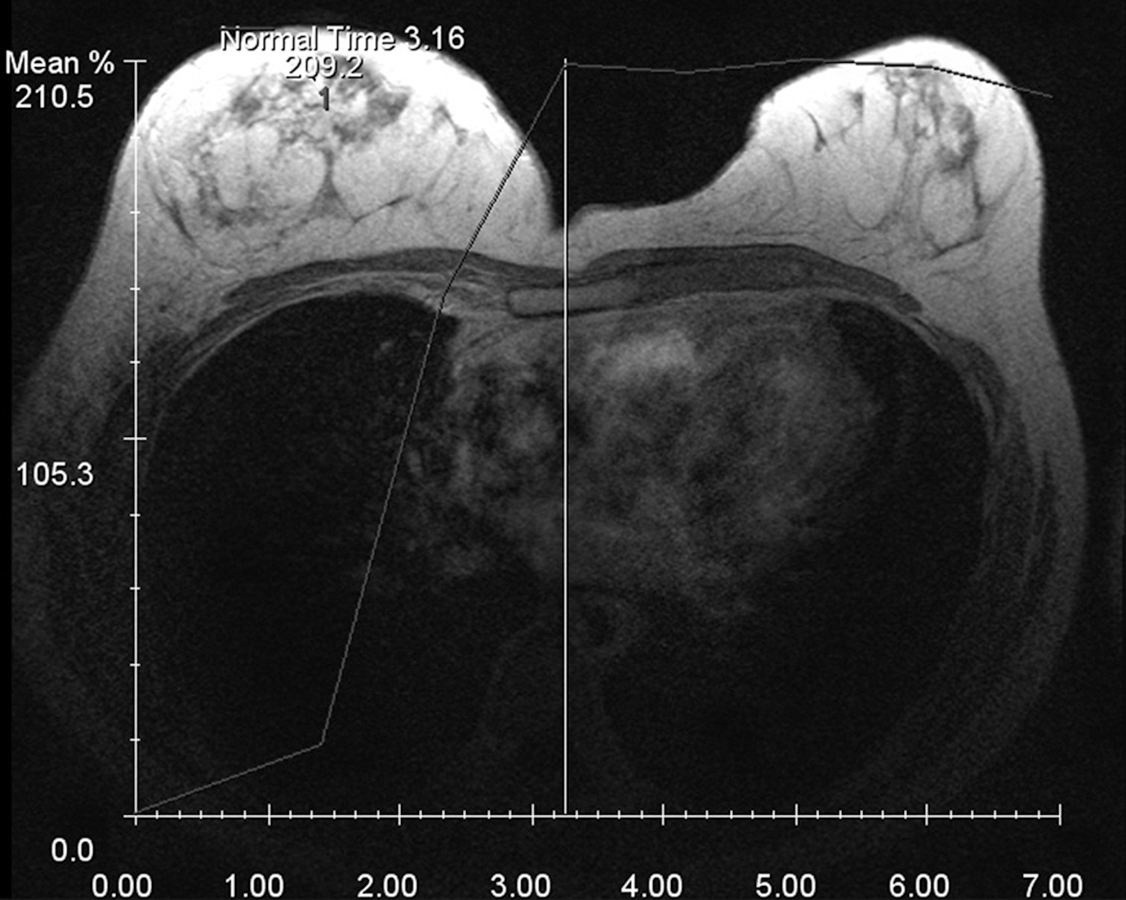
Figure 3. Plateau type time enhancement curve in a DCIS

Combination of morphologic and kinetic patterns – the “composite” parameters differ significantly (P>0.05), although the most frequent morpho-kinetic category included only six lesions (26.1%) presented as focal mass-like enhancement with plateau curve type.

## 5. Discussion

In this study, DCIS appeared as non-mass-like lesions in more than half of the lesions (12 lesions, 52.17%) compared to 10 mass lesions (43.48%) and only one focal lesion (4.35%). The differences in the lesion types proved to be statistically significant (P<0.05) that corresponds to the results of the published studies. The statistical difference between the proposed patterns was significant (P <0.05) with eight lesions (35%) having focal enhancement, as opposed to six segmental lesions (26%): regional and triangular. Our data slightly disagreed with those published by Neubauer et al. who stated that the most frequent morphological pattern included as high as 82% of segmental enhancement, as opposed to 72% of dotted or granular pattern (18). Jansen et al. stated that the most frequent morphology included non-mass-like lesions that correspond to our conclusions, although the enhancement pattern (clumped or heterogeneous enhancement) was in segmental or linear distribution that partly differs from our findings with no predominant distribution pattern (16). The kinetic patterns included three types of curves; plateau type, occurring most frequently (13 lesions, 56.5%); followed by the continuous type (six lesions, 26%) and the washout type (four lesions, 17.4%). In our series, the difference between the curve types was not statistically significant (P>0.05); however, the curve types suspicious of malignancy–plateau and washout–were found in almost 74% of the lesions. Our results were similar to those published by Neubauer et al. who concluded that 62% of all tumors comprised either plateau or washout curve type (18). We combined the morphologic and kinetic parameters in order to define the most frequent morpho-kinetic pattern. However, there was no statistically significant difference among the patterns, in which focal enhancement with plateau curve type kinetics appeared in six lesions (26.1%), followed by segmental enhancement with plateau curve type. Recognition of dominant morphologic, kinetic and morpho-kinetic characteristics of DCIS would lead to improvement of detection of early-stage breast carcinoma. Non-mass-like lesions, lesions with focal or segmental distribution with “plateau” kinetics appear to be the most frequent confirmed DCIS lesions seen in MRI.
